# Anti-HBV efficacy of combined siRNAs targeting viral gene and heat shock cognate 70

**DOI:** 10.1186/1743-422X-9-275

**Published:** 2012-11-16

**Authors:** Zhongqi Bian, An Xiao, Mingmei Cao, Mingqiu Liu, Shuang Liu, Ye Jiao, Weiyao Yan, Zhongtian Qi, Zhaoxin Zheng

**Affiliations:** 1 Center for Infectious Diseases, Kunming General Hospital, PLA, 212 Daguan Rd, Kunming, 650032, P. R. China; 2 State Key Laboratory of Genetic Engineering, Institute of Genetics, School of Life Science, Fudan University, 220 Handan Rd, Shanghai, 200433, P. R. China; 3 Department of Microbiology, Shanghai Key Laboratory of Medical Biodefense, Second Military Medical University, 800 Xiangyin Rd, Shanghai, 200433, P. R. China

**Keywords:** Hepatitis B virus, RNA interference, Short hairpin RNA, Heat shock cognate 70, Combinational RNAi

## Abstract

**Background:**

Hepatitis B virus (HBV) infection is a major health concern with more than two billion individuals currently infected worldwide. Because of the limited effectiveness of existing vaccines and drugs, development of novel antiviral strategies is urgently needed. Heat stress cognate 70 (Hsc70) is an ATP-binding protein of the heat stress protein 70 family. Hsc70 has been found to be required for HBV DNA replication. Here we report, for the first time, that combined siRNAs targeting viral gene and siHsc70 are highly effective in suppressing ongoing HBV expression and replication.

**Methods:**

We constructed two plasmids (S1 and S2) expressing short hairpin RNAs (shRNAs) targeting surface open reading frame of HBV(HBVS) and one plasmid expressing shRNA targeting Hsc70 (siHsc70), and we used the EGFP-specific siRNA plasmid (siEGFP) as we had previously described. First, we evaluated the gene-silencing efficacy of both shRNAs using an enhanced green fluorescent protein (EGFP) reporter system and flow cytometry in HEK293 and T98G cells. Then, the antiviral potencies of HBV-specific siRNA (siHBV) in combination with siHsc70 in HepG2.2.15 cells were investigated. Moreover, type I IFN and TNF-α induction were measured by quantitative real-time PCR and ELISA.

**Results:**

Cotransfection of either S1 or S2 with an EGFP plasmid produced an 80%–90% reduction in EGFP signal relative to the control. This combinational RNAi effectively and specifically inhibited HBV protein, mRNA and HBV DNA, resulting in up to a 3.36 log_10_ reduction in HBV load in the HepG2.2.15 cell culture supernatants. The combined siRNAs were more potent than siHBV or siHsc70 used separately, and this approach can enhance potency in suppressing ongoing viral gene expression and replication in HepG2.2.15 cells while forestalling escape by mutant HBV. The antiviral synergy of siHBV used in combination with siHsc70 produced no cytotoxicity and induced no production of IFN-α, IFN-β and TNF-α in transfected cells.

**Conclusions:**

Our combinational RNAi was sequence-specific, effective against wild-type and mutant drug-resistant HBV strains, without triggering interferon response or producing any side effects. These findings indicate that combinational RNAi has tremendous promise for developing innovative therapy against viral infection.

## Background

More than 350 million of about 2 billion people in the world exposed to the hepatitis B virus (HBV) are chronically infected and at serious risk of developing liver failure, cirrhosis, and hepatocellular carcinoma (HCC) [1-3]. About 75% of them reside in the Asia-Pacific region [4], particularly in Asian endemic areas such as China. Each year, ~600,000 HBV-related deaths occur worldwide [5]. Approved therapies for chronic hepatitis B include interferon (IFN)-alfa and nucleos(t)ide analogues, but rarely eliminate the virus [6]. HBV persists by establishing HBV covalently closed circular DNA (ccc DNA) in hepatocytes, which nuclear transcription template continues to initiate new HBV replication cycle even after serologic clearance. Long-term treatment in most cases bears the risk of adverse side-effects and mutant drug-resistant HBV strains. Therefore, combinational strategies for treating HBV from different angles are urgently needed. In infected hepatocytes, HBV produces four major classes of messenger RNAs (mRNAs). A 3.5 kb pregenomic RNA is reverse-transcribed into new HBV genomes and serves as mRNA for translating the viral core and polymerase proteins. A minimally longer RNA encodes the secretory hepatitis B e antigen (HBeAg). RNAs (2.4 and 2.1 kb) serve as mRNA for viral envelope proteins L, M, and S. From 0.7 kb RNA the HBV X protein is translated (Additional file 1: Figure S1).

RNA interference (RNAi) is a sequence-specific post-transcriptional gene-silencing molecular mechanism that was first discovered in *Caenorhabditis elegans*[7]. RNAi process is initiated by an RNase III enzyme known as Dicer that processes dsRNAs into 21–25 nt small interfering RNAs (siRNAs). Subsequently, these siRNAs are unwound, and one strand is preferentially loaded into the RNA-induced silencing complex (RISC). The loaded single-stranded RNA, called the antisense guide strand, then directly targets the complementary mRNA for cleavage or transcriptional repression and degradation, with the other strand — the ‘passenger’ — being degraded [8-11]. RNAi can be induced via synthetic siRNAs [12] or DNA vectors for intracellular expression of short hairpin RNAs (shRNAs) [11,13]. RNAi-based gene silencing approaches have been demonstrated in humans, and ongoing clinical trials hold promise for treating fatal disorders or providing alternatives to traditional small molecule therapies [11]. There have been many studies reporting on highly effective RNAi-mediated silencing of human immunodeficiency virus type 1(HIV-1) [14-16], HBV [17-21], hepatitis C virus(HCV) [22,23], hepatitis E virus [24], influenza virus [25], SARS-CoV [26] and Ebola filoviruses [27] in cell culture and in vivo. In addition, some siRNAs have been found to be potent sequence-dependent inductions of the mammalian innate immune response [28-31]; they are also reported to be possessed of bifunctional antiviral molecules that induce production of type I IFNs in the liver and target HBV to inhibit viral replication [21]. However, technical challenges remain, such as how to deliver siRNAs specifically into target genes in a therapeutically acceptable way [11,32,33] while avoiding adverse side effects [34].

HBV strains rely heavily on host cell machinery to complete their life cycles. A number of host proteins have been found to be crucial for HBV/ HCV replication [35-39]. Hsc70 (or HspA8) is an ATP-binding protein of the Hsp70 family [40]. This host protein has been found to be required for the reverse transcription process in HBV DNA replication [41,42]. Host proteins can also be targeted, for example siRNAs directed to both diacylglycerol acyltransferase 1 (DGAT1) and the host gene product polo-like kinase 1 (PLK1) can reduce HCV production [43,44]. Notably, Nakagawa *et al*. and Liu *et al*. [38,45,46] have demonstrated that down-regulation of Hsp70 or Hsp90 by siRNA significantly inhibited HBV/HCV production with no cytotoxicity, cellular proliferation or apoptosis. A current focus of several laboratories is to use miRNA or shRNA expression methods to target more than one viral transcript [47-49]. As chronic hepatitis contributes significantly to hepatocellular carcinoma pathogenesis, this further stimulates interest in new HBV therapies to reduce disease burden.

We [33,50-57] previously showed sequence-specific inhibition by RNAi of HBV/HCV/FMDV and EGFP *in vitro* and *in vivo*. Recently we [58] reported that Japanese encephalitis virus infection in Huh7 cells presupposes the association of Hsp70 with lipid rafts, and HBV has been demonstrated to promote tumor cell invasion by a mechanism involving the up-regulation of heat shock protein 90 (Hsp90) [59]. There has so far been no report on using the siHBV and endogenous Hsc70 targeting (siHsc70) combination to treat HBV. Here we demonstrate that siHBV in combination with siHsc70 in HepG2.2.15 cells is an innovative effective approach to treating HBV without triggering innate immune response, and that their antiviral synergy produces no cytotoxicity and does not affect cell viability or proliferation.

## Results

### siRNAs effectively suppressed the expression of fusion EGFP in HEK293 and T98G cells

**Figure 1:**
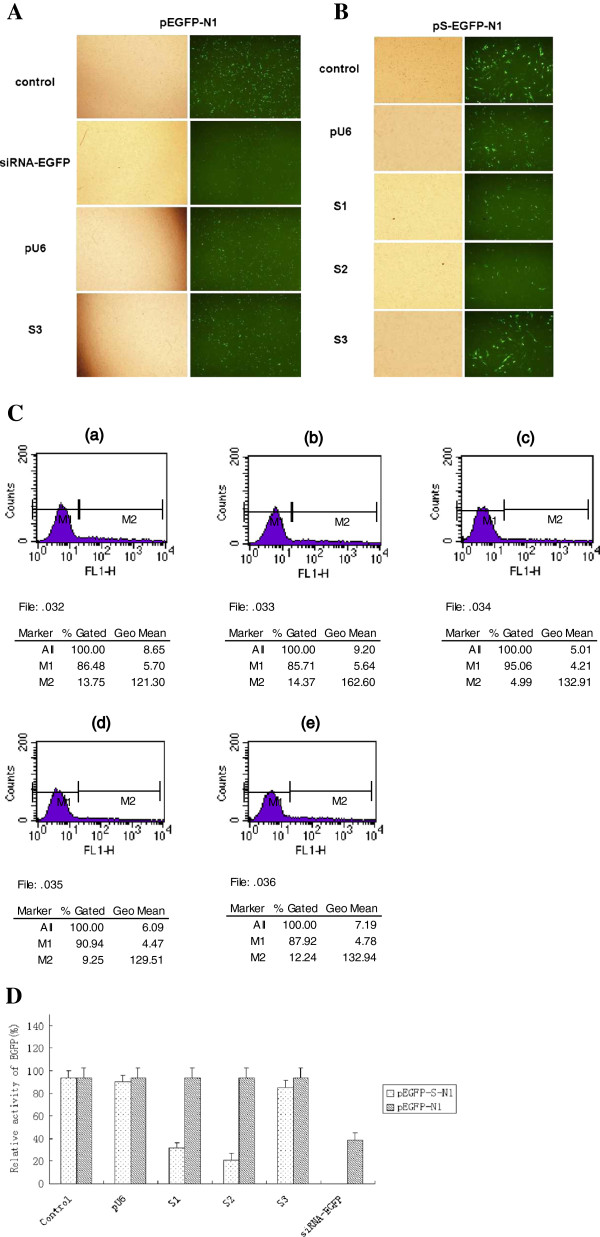
**Effect of siRNAs on the expression of HBV surface open reading frame in HEK293 and T98G cells. (A)** As shown in Additional file 2: Figure S2. **(B)** Fluorescence micrographs of cells transfected with reporter plasmids and cotransfected with either the corresponding or non-corresponding siRNA with Lipofectamine ^TM^ 2000 (Invitrogen). At 24 hrs after transfection, the cells were observed with an Olympus BH-2 microscope, and representative bright-field images (left column) and relative fluorescent-field images (right column) were recorded by tenfold amplification. **(C)** Flow cytometry analysis of siRNA-mediated gene silencing of EGFP. Level of EGFP expression in cells cotransfected with (a) pEGFP-N1 vector; ( b) pS-EGFP-N1 and pU6; (c) pS-EGFP-N1 and S1; (d) pS-EGFP-N1 and S2; (e) pS-EGFP-N1 and S3 (heterologous siRNA). **(D)** Cells were analyzed for EGFP expression by fluorescence-activated cell sorting and the level of fluorescence relative to the control was quantitated. The mean fluorescence intensity of control siRNA was taken as 100% and adopted as control. Data represent means±SD from three independent experiments were performed in triplicate.*Significant differences compared to the heterologous siRNA control: Student’s t-test; P < 0.05.

The siRNAs targeted to the conserved regions of HBV genome were generated by intracellular Dicer enzyme, as depicted in Additional file 1: Figure S1A. To identify an effective inhibitory efficacy of siRNAs, the DNA cassettes of these regions were inserted into the 5’ end of enhanced green fluorescent protein (EGFP) gene to construct reporter plasmids (Additional file 1: Figure S1B).The reporter plasmids were transfected into HEK293 and T98G cells with either the homologous siRNAs or the heterologous siRNAs.The number of EGFP-expressing cell was examined by fluorescent microscope 24 h after transfection so as to the verification of an RNAi-mediated mechanism. We found that the number of cells in visible light were comparable in cells transfected with homologous siRNAs relative to cells transfected with heterologous siRNAs or non-transfected cells. This indicates that siEGFP does not vitiate cellular growth and survival. After green fluorescent light was put into action, it could be seen that in comparison with the other groups, the expressivity of EGFP decreased markedly in the siEGFP group. This indicates that in addition to impacting post-transcriptional translation, siEGFP exercised its specific, gene-silencing effect on the EGFP, resulting in cessation of EGFP expression (Figure 1A, B and Additional file 2: Figure S2A). The expression of EGFP was determined by flow cytometry, and the same conclusion was reached by making a comparison of the different groups (Figure 1C, D and Additional file 2 Figure S2B). Statistically significant differences existed between the siEGFP group and the controls (P < 0.05). This was further confirmed with Western blotting by assessing siEGFP inhibition of the expression of EGFP fusion protein and produced the same results (data not shown). These results demonstrate that shRNAs have been generated from siRNA-expressing plasmid and efficiently processed by intracellular Dicer enzyme turn into corresponding siRNAs as RNAi on the target gene (Figure 1). Cotransfection of either S1 or S2 with a reporter plasmid produced an 80%–90% reduction in EGFP signal relative to the control. Fluorescence-activated cell sorting demonstrated that the levels of inhibition mediated by the siRNAs were similar among the different experiment groups and significantly higher than the control group (cotransfection with heterologous siRNAs or without siRNAs). To further detect inhibitory effectiveness, cells were collected 48 h after transfection and the inhibitory potency of siRNAs was assessed by quantitative real-time PCR and reverse transcription-PCR (RT-PCR) assay. The level of target RNAs was significantly reduced in cells transfected with homologous siRNAs, and the specific amplification of RT-PCR products confirmed by Melt-curve analysis (data not shown). The inhibition of EGFP and S mRNA expression were also demonstrated by RT-PCR analysis [33,50,56]. The correct transcription of EGFP and S was confirmed by sequencing of RT-PCR products. The results suggested that the siRNAs generated by intracellular transcription could effectively and specifically inhibit the expression of HBV in transfected cells.

### Synergistic inhibition of HBV protein expression by siHBV in combination with siHsc70 in HepG2.2.15 cells

**Figure 2:**
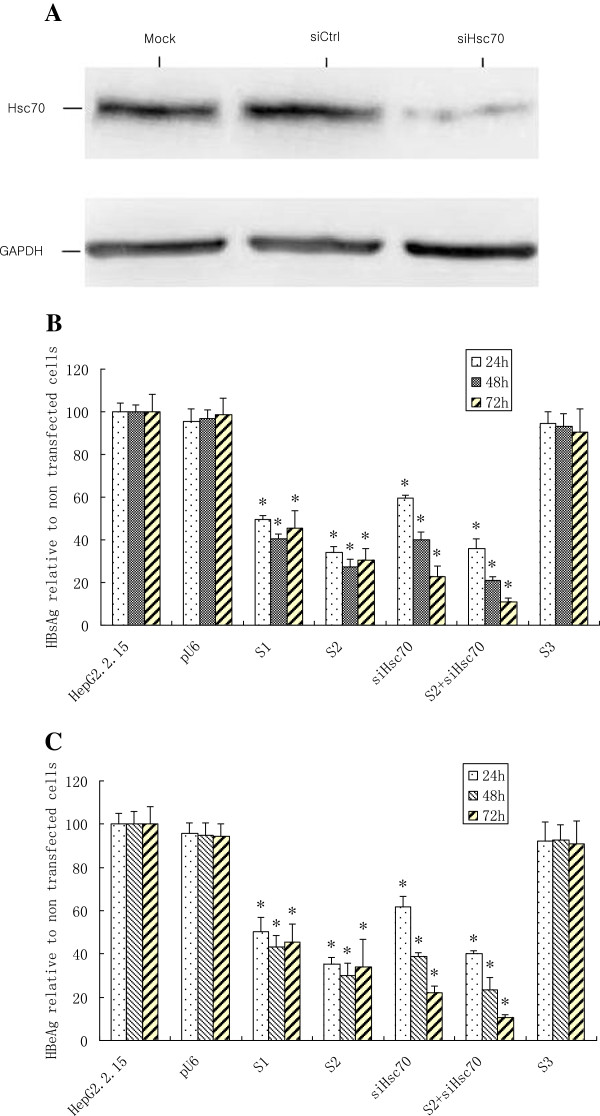
**Synergistic inhibition of HBV protein expression by siRNA targeting viral gene in combination with siHsc70 in HepG2.2.15 cells. (A)** The expression of Hsc70 in HepG2.2.15 cells transfected with siHsc70 or siCtrl after 72 hrs was identified by Western blotting with anti-Hsc70. GAPDH expression was used as an internal control. **(B)** The reduction of HBsAg expression. HepG2.2.15 cells transfected with pU6, S1, S2, siHsc70, S2 with siHsc70, and also the heterologous S3. **(C)** The reduction of HBeAg expression. HepG2.2.15 cells transfected with pU6, S1, S2, siHsc70, S2 with siHsc70, and also the heterologous S3. Data represent means±SD from three independent experiments were performed in triplicate.*Significant differences compared to the heterologous siRNA control: Student’s t-test; P < 0.05.

To determine the knockdown efficacy of shRNA expression cassettes that target the HBVS when used alone or in combination with a hairpin expression cassette that targeted the endogenous Hsc70, the amount of HBsAg and HBeAg in the cell culture supernatant was determined using ELISA at different time points after transfection. As depicted in Figure 2B and C, the S1 and S2 can independently and significantly inhibit HBsAg and HBeAg 48 h after transfection. The HBsAg was reduced 60.7% by S1 and 72.7% by S2, while the HBeAg decreased 56.9% with S1 and 69.8% with S2, as compared with the heterologous siRNA control (P < 0.05). As shown in Figure 2A, the expression of Hsc70 was abrogated by siHsc70, as compared with control. The reductions of HBsAg and HBeAg were about 60.2% and 61.2% individually by siHsc70 at 48 h after transfection, while the combination of S2 and siHsc70 markedly inhibited 89.1% of HBsAg and 89.6% of HBeAg individually in the supernatants of HepG2.2.15 cells 72 h after tansfection with S2 and siHsc70, as compared with the homologous siRNA or the heterologous siRNA (P < 0.05). The results indicated that the combined siRNAs were more potent than the siHBV or siHsc70 used separately.

### Specific inhibition of HBVS mRNA by siHBV in combination with siHsc70 in HepG2.2.15 cells

**Figure 3:**
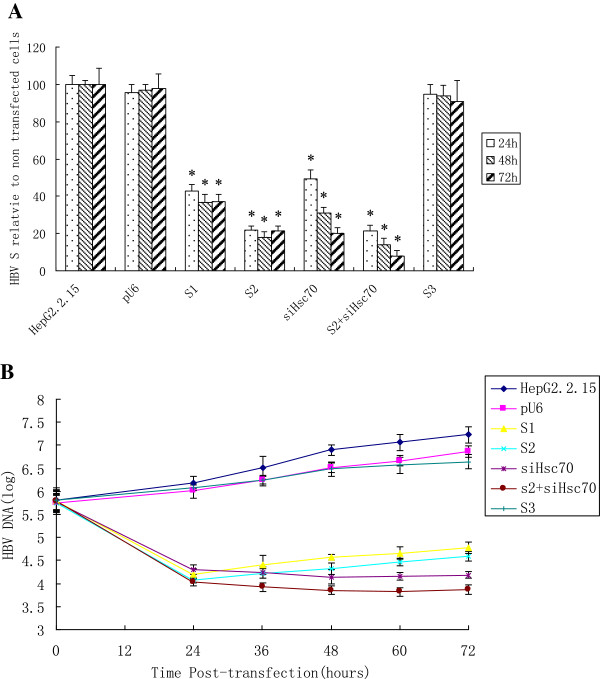
**Specific inhibition of HBV genes by siRNA targeting viral gene in combination with siHsc70 in HepG2.2.15 cells.** (**A**) The inhibition of HBVS mRNA by siRNAs at different time points. HepG2.2.15 cells were transfected with pU6, S1, S2, siHsc70, S2 with siHsc70, and also the heterologous S3. Data represent means±SD from three independent experiments were performed in triplicate. *Significant differences compared to the heterologous siRNA control: Student’s t-test; P < 0.05. **(B)** Inhibition of HBV DNA by siRNAs in cell culture supernatants. HepG2.2.15 cells were transfected with pU6, S1, S2, siHsc70, S2 with siHsc70, and also the heterologous S3. Data represent means±SD from three independent experiments were performed in triplicate.

As depicted in Figure 3A, the S1, S2, and siHsc70 could effectively and specifically inhibit the expression of HBVS gene 24 h after transfection, with reduction of HBVS mRNA by 63.4%, 72.2% and 69.2% respectively 48 h after transfection, whereas the heterologous siRNA control revealed no significant effects on HBVS mRNA in HepG2.2.15 cells. When the S2 was used in combination with siHsc70, their synergistic inhibition of HBVS mRNA expression grew markedly to 86.3%, indicating that the combined siRNAs were more potent than S2 or siHsc70 used individually. The results showed that combinational RNAi effectively and specifically inhibited the expression of HBVS mRNA.

### Specific inhibition of HBV DNA by siHBV in combination with siHsc70 in HepG2.2.15 cells

As depicted in Figure 3B, as compared with the controls, HBV DNA decreased distinctly in cell culture supernatants 24 h after transfection with plasmids S1, S2, siHsc70 or S2 and siHsc70 respectively, with their inhibitory effects most noticeable 72 h after transfection. The HBV DNA copies in S1, S2 or siHsc70 treated cells was found to have been reduced by 2.44 log_10_, 2.64 log_10_ and 3.04 log_10_ respectively 72 h after transfection, while the combination of siHsc70 and S2 produced a 3.36 log_10_ decrease in HBV load of the cell culture supernatants. The controls did not showed a significant reduction from the heterologous siRNA at any time point. Thus siRNAs of the HBV genome targeting and endogenous gene targeting combination were effective and specific, and resulted in an overall reduction of virus load, which indicated that the combined siRNAs were more potent than the siHBV or siHsc70 used separately.

### Silencing Hsc70 does not affect cell viability

Hsc70 are highly-conserved essential stress proteins. Therefore, we next investigated whether gene-silencing of host protein affected cell viability and consequently viral production. An MTT assay, measured at A570, determined that siRNA-mediated silencing of Hsc70 had no significant effect on cellular proliferation (or cytotoxicity). A GAPDH Western blotting was used as an internal control to verify that equivalent numbers of cells were used in each assay. These results indicate that siRNA-mediated gene silence of Hsc70 does not affect cell viability.

### Effects of siRNAs on IFN-α, IFN-β, TNF-α in HEK293, T98G cells and HepG2.2.15 cells

**Figure 4:**
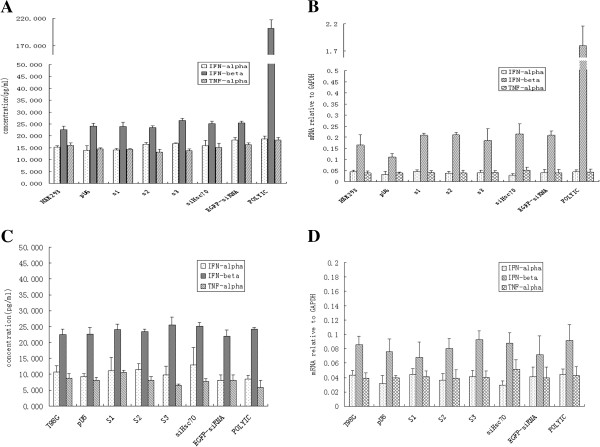
**(A, B, C, D) Effects of siRNAs on IFN-α, IFN-β, TNF-α in HEK293 and HepG2.2.15 cells. (A)** HEK293 and HepG2.2.15 cells were respectively transfected with pU6, S1, S2, S3, siHsc70, EGFP-siRNA and the positive-control poly (I:C). The concentrations of IFN-α, IFN-β and TNF-α in cell culture supernatants from transfected cells were determined by ELISA assay. **(B)** The effects of siRNAs on the mRNA concentrations of IFN-α, IFN-β, and TNF-α in HEK293 cells. HEK293 and HepG2.2.15 cells were respectively transfected with pU6, S1, S2, S3, siHsc70, EGFP-siRNA and the positive-control poly (I:C). The mRNA concentrations of IFN-α, IFN-β and TNF-α were examined by RT-PCR amplification with the SYBR RT-PCR Kit. Data represent means±SD from three independent experiments were performed in triplicate. **(C)** T98G cells were transfected with pU6, S1, S2, S3, siHsc70, EGFP-siRNA and the positive-control poly (I:C). The concentrations of IFN-α, IFN-β and TNF-α in cell culture supernatants from transfected cells were determined by ELISA assay. **(D)** The effects of siRNAs on the mRNA concentrations of IFN-α, IFN-β, and TNF-α in HEK293 cells. HEK293 cells were transfected with pU6, S1, S2, S3, siHsc70, EGFP-siRNA and the positive-control poly (I:C). The mRNA concentrations of IFN-α, IFN-β and TNF-α were examined by RT-PCR amplification with the SYBR RT-PCR Kit. Data represent means±SD from three independent experiments were performed in triplicate.

We investigated whether the IFN pathway induction could be stimulated in siRNA transfected cells as reported in earlier studies [28-31]. The results showed that positive-control poly (I:C) caused intense IFN-β secretion in HEK293 and HepG2.2.15 cells, while the siRNAs induced no production of IFN-α, IFN-β and TNF-α in transfected cells (Figure 4A and B), and IFN-α, IFN-β and TNF-α mRNA concentrations were not detected in T98G cells as measured by ELISA and RT- PCR (Figure 4C and D). By combining with receptor TLR3, the IFN-β response generated by the poly (I:C) as IFN activated the downstream signal pathway to induce releases of type I IFN. As can be seen in Figure 4, the poly (I:C) induced strong IFN response in HEK293 cells, resulting in massive IFN-β expression and no IFN-α or TNF-α expression. A comparison with these cells not transfected with any plasmid revealed that the impact of S1, S2, S3, siHsc70, and siEGFP on production of type I IFN and TNF-α in transfected cells was negligible or no immunostimulation. Taken together, we showed that the poly (I:C) could not induce IFN response in T98G cells, which indicates that expression by receptor TLR3 in T98G cells were little to none. We found that the siRNAs tested did not induce the innate IFN response whereas the poly (I:C) control was a good stimulator.

## Discussion

In this study, we showed for the first time that combined siRNAs targeting the genes of HBVS and siHsc70 is specific and highly effective in suppressing ongoing viral gene expression and replication in HepG2.2.15 cells while forestalling escape by mutant HBV. The combined siRNAs were more potent than siHBV or siHsc70 used separately, without triggering IFN response or producing any side effects. In agreement with research by Liu *et al*. [45], our work demonstrates that the antiviral synergy of siHBV used in combination with siHsc70 produces no cytotoxicity and does not affect cell viability. Recently, modular trimeric Pol II expression cassettes comprising miRNA shuttles have been used successfully to generate multiplexed anti-HBV RNAi activators [60]. We constructed plasmids siHBV and siHsc70, and employed an HBV genes targeting and endogenous Hsc70 genes targeting combination, while Ely *et al*. [60] reported that they constructed miR expression plasmids, and generated cassettes using primiR-31-, pri-miR-30a- and pri-miR-122-derived modules, which were combined as trimers and expressed from a Pol II promoter. The plasmids we constructed and the combinational approach we adopted are markedly different from those Ely *et al*. did. Interestingly, their study and ours produced the same results [60].

Molecular chaperones were initially discovered as mediators of the cellular heat shock response [61], and subsequent studies have demonstrated promiscuous functions for these proteins, including those related to both cancer- and virus-associated pathogenesis [62]. The heat stress protein 70 (Hsp70) family is composed of highly conserved proteins, including Hsc70, Hsp70, heat shock protein 90 (Hsp90) , GRP75 and GRP88. Hsc70 (or Hsp90) is a well-characterized, multifunctional molecular chaperone involved in regulation of signal transduction, transcriptional activation, oncogenic protein stabilization, and neovascularization—pathogenic elements relevant to viral cancer pathogenesis [62]. Despite functioning primarily as cytoplasmic chaperones, these family members are found on the surfaces of various cell types, such as tumor cells, neural stem cells, spermatogenic cells, epidermal cells, arterial smooth muscle cells, monocytes and B-cells [63]. Hsp’s acting as virus receptors on cell surfaces have been described in some viral infections, e.g., rotaviruses [64], human T lymphotropic virus Type 1 [65], coxsackievirus A9 [66] and DENV [67]. The host protein plays a critical role in various stages of the virus life cycle from entry, replication and assembly to egress of the virus particles. Hsc70 has been found to play a role in the life cycles of a variety of RNA and DNA viruses [68]. Inhibitor for Hsc70 mRNA/protein expression could inhibit HBV/HCV replication efficiently [69,70]. Medications that inhibit Hsc70 expression are effective in suppressing infection by wild-type viral strains and effective against viral strains resistant to lamivudine and suchlike medications, thus effectively obviating HBV resistance to drugs. Unlike those that target viral genes or enzymes, siRNA specific to host Hsc70 genes would be effective against wild-type and mutant drug-resistant HBV strains. By suppressing Hsc70 mRNA expression in host cells, siHsc70 can markedly suppress HBV replication [51,52]; drugs targeted at Hsc70 are active against wild-type HBV while simultaneously suppressing replication of viral strains resistant to lamivudine, entecavir, telbivudine and kindred drugs [69,70]. Because all HBV RNAs share common 3’ sequences, they may be targeted by a single siRNA. Replication of the virus is susceptible to RNAi-mediated inhibition and unlike HIV-1 or HCV, HBV genome is not prone to mutation with escape from silencing by antiviral shRNAs. This is primarily because HBV has highly compact genome with overlapping reading frames (Additional file 1: Figure S1C). These factors might explain, at least in part, why these two HBV-specific siRNAs have the highest efficacy. The two plasmids S1 and S2 we constructed were targeted at the conserved region sequences in HBV genome subtype ayw, which was identical with the virus we had previously reported (GenBank accession number AY 517489 and AY 517619) [71]. The plasmids S1 and S2 are HBV-specific siRNAs (siHBV) and directly knock down transcript of HBVS; Hsc70 is a novel potential target for developing drugs against HBV, and siHsc70 indirectly inhibits HBV replication and expression by virtue of inhibiting host proteins involved in HBV infection. Their target sites and functioning mechanisms are different, but their antiviral effects are the same and can work in synergy. siRNAs directly targeting HBV genes are liable to forfeit their inhibitory efficacy on account of HBV genes mutating under selective pressure. The target site of siHsc70 is on Hsc70, a host protein of remarkable stability, not subject to mutation under normal circumstances. In terms of inhibiting the expression of HBVS and e proteins, siHsc70 used in conjunction with S2 is more potent than S2 or siHsc70 used in isolation by 6.3%, 6.9%, 18.8%, and 15.5% respectively. Most importantly, our results showed that combinational RNAi markedly inhibited HBV protein, mRNA and HBV DNA, resulting in up to a 3.36 log_10_ reduction in HBV load in the HepG2.2.15 cell culture supernatants. Hsc70 gene knockout generates no abnormality in mice, proving its safety after inhibition of Hsc70 [45]. Therefore, The combined siRNAs exercising their effect on Hsc70 can make up for flaws with S2 and thus can not only inhibit wild-type HBV, but also can inhibit the infectiousness of mutant strains as well. S3 did not elicit significant inhibition of HBV, suggesting that siRNAs mediated significant reductions in a specific target mRNA, and not a general down-regulation resulting from activation of the dsRNA-activated protein kinase R (PKR), which could induce inhibition of protein translation in a non-sequence specific manner. As is well known, RNAi acts as a natural antiviral defense mechanism in plants, especially against RNA viruses. Mammalian cells were originally presumed to be unlikely to inherently possess an active RNA-silencing machinery, but primarily to induce a nonspecific, interferon-mediated antiviral response mediated by dsRNA, especially by viral long dsRNA. Some of the siRNA sequences tested showed a vigorous IFN-a response. Reportedly, in many cases transfection of siRNAs (>35nt), even siRNAs (<23nt) results in IFN-mediated activation of the Jak–Stat pathway and global upregulation of IFN-stimulated genes (ISGs), which was mediated by PKR and Toll-like receptors (TLRs). Therefore, it may be that the innate immune system can recognize immunostimulatory RNA motifs within both single-stranded RNA (ssRNA) and double-stranded RNA(dsRNA) via TLR7 or TLR8 [28-30]. Further work is required to define the core RNA motifs underlying immune recognition of siRNA. TLR7 and TLR8 have been shown to be restricted to cells of the innate immune system. Previous studies [30,31] reported the activation of the IFN pathway induction in response to transcribed siRNAs in HEK293 and T98G cells. The induction of the IFN and its effect on cellular signalling pathways indicates that siRNAs have broad effects beyond the selective silencing of homologous target genes when introduced into cells.

The present study indicates that vector-based siRNA without sequence of 5’ UGUGU 3’ and having lower ratios of UG content minimize side effects on the innate immune system [28]. Still, there have been studies reporting different results, which indicates that the mechanism in inducing innate IFN response is not reducible to length and sequence dependence, and which compels conjectures over the possible role that RNA structures might play. As regards whether and how siRNA can induce innate immune response in mammalian cells, different studies have produced different results. At present, there is no clear understanding of the mechanisms that determine the gene-silencing efficiency of a given siRNA. Viruses have evolved mechanisms to suppress or escape from RNAi. Strategies aimed at providing rapid, efficient protection against HBV have to surmount a major challenge—that is, acute infection by the virus. Presumably siRNAs elicit an antiviral response in cells within 24 h, promising development of emergency RNAi vaccines to prevent virus disease, especially those capable of producing prompt prophylactic or therapeutic effects against HBV. In addition to differences in the efficiency of gene silencing due to differences in the structures of siRNAs and of their targets, it is suggested that other layers of complexity be addressed, including the extent of conservation of the RNAi machinery and its activity in many different mammalian cell types. There are still many hurdles to overcome before RNAi is used to treat chronic HBV, particularly relating to (i) appropriate delivery of RNAi molecules and (ii) continual presence of cccDNA in the hepatocyte nucleus which provides a constant source of viral mRNAs and pregenome. However, our findings suggest that combinational RNAi is worth pursuing in developing new approaches to treat and cure the 400 million people worldwide living with chronic HBV infection. Further studies should be conducted with mammals in order to obtain unanticipated results regarding the aforementioned issues and therapeutic applications of the combinational RNAi system.

In terms of viral clearance, our results highlight the interest of a combined therapy. Such an approach is capable of reducing the drug toxicity and preventing the antiviral compound drug resistance, thus minimizing chances of viral escape. The innovative combinational RNAi approach to treating HBV is fundamentally distinct in that it inhibits viral expression as well as inhibition of viral replication. Weakness in host immune response associated with chronic HBV infection makes it virtually impossible to achieve sustained, complete viral clearance, even after long-term suppression of HBV DNA replication with lamivudine or telbivudine, presumably because these therapies fail to reduce high viral antigenemia believed to mitigate T cell response in chronically infected patients and thereby help HBV avoid immune clearance. In contrast, reducing viral antigen levels attained after combinational RNAi-mediated degradation of viral RNA transcripts (pregenomic RNA, precore/ HBsAg mRNAs) may relieve the negative effect of chronic antigen stimulation on T cell response and facilitate recovery of that immune response. Since certain viral proteins, as the HBeAg, have been reported to suppress innate immune responses that inhibit viral persistence, reduction of viral protein synthesis could be an additional benefit of RNAi -mediated therapy.

In little more than a decade, RNAi discovery has led to understanding the molecular processes responsible for small RNA biogenesis and function, as well as to developing reagents that utilize the power of the RNAi pathway. Notwithstanding the numerous hurdles for translating these technologies into therapy, such as important considerations for therapeutic RNAi that gene silencing approaches rarely remove 100% of a transcript, that off-target silencing can occur and that each target organ, cell type and target transcript presents unique challenges. Promising early clinical results warrant guarded optimism.

## Conclusion

In summary, we have demonstrated for the first time that combinational RNAi is specific and highly effective in suppressing ongoing viral gene expression and replication in HepG2.2.15 cells while forestalling escape by mutant HBV. The combined siRNAs were more potent than HBV siRNA or siHsc70 used separately, without triggering interferon response or producing any side effects. This approach markedly inhibited HBV protein, mRNA and HBV DNA, resulting in up to a 3.36 log_10_ reduction in HBV load in the HepG2.2.15 cell culture supernatants. The antiviral synergy of siHBV in combination with siHsc70 produced no cytotoxicity and induced no production of IFN-α, IFN-β and TNF-α in transfected cells. Although this approach should prove to be an effective therapy against HBV, clinical application remains to be further tested and examined. Nonetheless, the data presented here justify continued explorations into this innovative combinational RNAi approach to treating HBV/HCV and HIV infection.

## Materials and methods

### Selection of target sequences

The reference sequences of the conserved regions of HBV genome were obtained from the National Center for Biotechnology Information (NCBI) website (http://www.ncbi.nlm.nih.gov) and compared with those of HBV by nucleotide (nt) BLAST. The genes and the regions of interest were essential during the life cycle of the virus and relatively conserved at the nucleotide sequences, as diagrammed in Additional file 1: Figure S1C.

HBV target sequences were chosen in regions overlapping the viral 3.5-kb, 2.4-kb, and 2.1-kb RNAs, according to the parameters indicated on the siRNA Target Finder web site (http://www.ambion.com). The 21 nt target sequences were selected as potential siRNA target sites based on the S gene targeted at conserved regions in the HBV genome originating from HepG2.2.15 cells (Stable HBV DNA-producing cell lines, subtype ayw, GenBank accession number U95551) [72]. Comparison of the HBV genome sequences (subtype ayw, GenBank accession number U95551) with HBV subtype ayr (GenBank accession number NC003977), adw(GenBank accession number EF103278), and adr (GenBank accession number AB299858) genome sequences by means of DNA STAR software MegAlign showed that the two 21-nt siRNAs targeting the HBVS gene were respectively homologous with subtype ayw 357 nt—377nt and 421nt—441nt. Through sequential analysis we found that the sequence homologous with S1 exhibited two mutant points—359 nt and 369 nt—in the four subtypes of HBV genome sequences, and that the sequence homologous with S2 had only one mutant point, 438nt (Additional file 3 Figure S3 and Additional file 4: Figure S4). In theory, siRNAs targeted at fewer mutant points in the HBV genome would exercise cross-inhibitory effects on various subtypes.

### Plasmid construction

We constructed two plasmids (S1 and S2) expressing shRNAs targeting S sequences of HBV from GenBank sequence data (accession number U95551) and one Hsc70-specific siRNA-expressing plasmid (siHsc70), and we used the control EGFP-specific siRNA plasmid ( siEGFP), as we had previously described methods [33,56]. The siHsc70 is identical in construction to two shRNA-expressing plasmids. Briefly, the mouse U6 promoter (pU6) was chemically synthesized from GenBank sequence data (accession number X06980) and cloned into the NdeI/EcoRI sites of pcDNA3.1B (−) vector (Invitrogen), replacing the human cytomegalovirus promoter (pCMV), to generate the parent vector pU6. Then, inverted repeats targeting the genome of HBV (Additional file 1: Figure S1C) were subcloned into pU6 at the EcoRI/HindIII sites, under the control of pU6 and a termination signal of five thymidines (Ts) (Additional file 1: Figure S1A). Plasmid S1 contains an inverted repeat corresponding to nt 201 to nt 221 of the DNA of HBVS, while plasmid S2 contains an inverted repeat corresponding to nt 265 to nt 285 of the DNA of HBVS. As a control for nonspecific effects, we used the shRNA-expressing plasmid S3 containing an inverted repeat of 21nt heterologous to the HBV genome, as confirmed by sequence analysis. To provide a reporting system for evaluating the gene-silencing efficacy of siRNAs, the DNA (681 nt) of HBVS was obtained by RT-PCR with DNA extracted from HepG2.2.15 cells as the template, using the primers 5’-CCGAAGCTTATGGAGAACATCACATCAGGA-3’(sense) and 5’-CGGCGAATTCAATGTATACCCAAAGACAAAAG-3’(antisense). Primers for retrotranscription of EGFP mRNA were 5’- GCCACCATGGTGAGCAAG-3’(sense) and 5’-CCCGCTTTACTTGTACAGC-3’ (antisense). RT-PCR products were further cloned into T-vector for sequencing [33,50]. The pS-EGFP-N1 was generated by cloning the DNA of HBV S into the EcoRI-BamHI sites of pEGFP-N1vector (Clontech, Palo Alto, CA) to form fusion EGFP (Additional file 1 Figure S1B) and reporter plasmids pS1-EGFP-N1, pS2-EGFP-N1, pS3-EGFP-N1 and psiEGFP-N1 were constructed respectively using previously reported methods [33,50,51,56]. The correct open reading frames confirmed by sequencing retained the fluorescent properties of the fusion protein.

### Cell culture and transfections

Three human cell lines, HepG2.2.15, HEK293, and T98G, were obtained from the ATCC. All cells were cultured in Dulbecco’s modified Eagle medium (DMEM, Invitrogen, CA)) supplemented with 10% fetal calf serum (Invitrogen), 100 units/ml penicillin/streptomycin, and 2% L-glutamine at 37°C with 5% CO_2_. HepG2.2.15 cells were additionally maintained in medium containing 380 μg/ml G418. The day before transfection, cells were seeded into 24-well plates (approximately 8 × 10^4^ cells/well) to achieve 60%-80% confluent cell monolayers. HepG2.2.15 cells were transfected with 0.8 μg of shRNA-expressing plasmids; HEK293 and T98G cells were transfected with reporter/target plasmids (0.6 μg) and either shRNA-expressing plasmids (0.3 μg) or pU6 (0.3 μg) or in combination, using Lipofectamine ^TM^ 2000 (Invitrogen) according to the protocol provided by the manufacturer. Transfection efficiency was calculated as the ratio between the number of viable transfected cells versus non-transfected cells. In our experiments, transfection efficiency was routinely above 90%.

### EGFP expression assay

To evaluate an effective inhibitory efficacy of siRNAs on expression of EGFP, cotransfected cells were identified as EGFP positive cells by fluorescence microscopy and flow cytometry ((Becton Dickinson, San Jose, CA). After an additional 24 h of incubation, cells were observed for the expression of EGFP on an Olympus BH-2 microscope and photographed using a Nikon E950 video camera at a magnification of × 10 with an exposure time of 4 s. Cells were further subjected to fluorescence-activated cell sorting, using previously described methods [33,50,51,56,59]. Silencing efficiencies were quantified by flow cytometry. Specific silencing of target genes was confirmed by RT-PCR and sequencing. Primers for retrotranscription of EGFP mRNA were 5’- GCCACCATGGTGAGCAAG-3’(sense) and 5’-CCCGCTTTACTTGTACAGC-3’ (antisense). RT-PCR products were cloned into T-vector for sequencing [33,50-52].

### Detection of Hsc70 protein by western blotting

Equal numbers of HepG2.2.15 cells transfected with siHsc70 or pU6-siRNA-Ctrl (heterologous to Hsc70 sequence) after 72 h were lysed in SDS sample buffer. As depicted in Figure 2A, cell lysates were separated by12% SDS-PAGE and the proteins transferred onto a polyvinylidene fluoride (PVDF) membranes (Millipore) using a Semi-Dry Electrophoretic Transfer System (Bio-Rad, CA) , and then probed with monoclonal antibodies specific for Hsc70 (clone 1B5; Stressgen) and glyceraldehyde-3-phosphate dehydrogenase (GAPDH), followed by incubation with horseradish peroxidase-labeled goat anti-mouse IgG as secondary antibody (Santa Cruz Biotechnology). Bound antibodies were detected by enhanced chemiluminescence (ECL) Plus Western blotting detection reagents (PerkinElmer Life Sciences). Hsc70 protein expression was also examined by flow cytometry using previously described methods [58,59].

### RNA preparation and RT-PCR

To detect HBV replication, total RNA was extracted from HepG2.2.15 cell cultures by Qiagen Rneasy Mini Kit, and subjected to quantitative real-time reverse transcription-PCR (RT-PCR). In brief, RT-PCR was performed in 24-well plates (Bio-Rad, Hercules, CA) in 20-μl reaction volumes containing the components of a SYBR RT-PCR Kit (Perfect Real Time; TaKaRa, Kyoto, Japan). The 20 μl reaction mixture contained 10 μl of SYBR master mix (2×), 0.4 μl of 0.2 μM forward primer and reverse primer respectively, 2.0 μl of a 1 μg RNA sample, and 7.2 μl of water. The cycle program consisted of 5°C for 30 min and 95°C for 10 s, followed by 40 cycles of 95°C for 5 s and 60°C for 20 s. Primers for retrotranscription of HBVS mRNA were 5’-TCACAATACCGCAGAGTC-3’(sense), and 5’-ACATCCAGCGATAACCAG-3’(antisense). To confirm specific amplification, melting-curve analysis of the RT-PCR products was performed according to the manufacturer’s protocol. Fluorescence was measured after each cycle and displayed graphically with iCycler iQ Real-Time PCR Detection System Software Version3.0A (Bio-Rad, Hercules, CA). GAPDH was used as an internal control, with 5’-CGGATTTGGTCGTATTGGG-3’(sense) and 5’- TCTCGCTCCTGGAAGATGG-3’(antisense) as primers. The cycle program employed was the same as the cycling parameters for Q-RT-PCR described above. Relative mRNA levels of HBVS were quantitated as the ratio of the HBVS gene product quantity to 1 ng GAPDH. RT-PCR products were cloned into T-vector for sequencing. The amounts of Hepatitis B surface antigen (HBsAg) and e antigen (HBeAg) in cell culture supernatants were quantified using commercial ELISA kits (Abbott Laboratories, North Chicago, IL). Assays were performed in triplicate independent experiments.

### Quantitation of HBV progeny DNA in culture supernatants

To measure the viral load, HBV DNA was extracted from HepG2.2.15 cell culture supernatants using a QIAamp DNA Mini Kit (Qiagen, Hilden, Germany), and the HBV DNA level was quantified using the Roche Diagnostics Cobas Taqman 48 (Meylan, France), which has a detection limit of 300 HBV DNA copies/ml. HBV genotypes were identified using S gene fragment sequencing.

### MTT assay for cell viability

The MTT cell viability assay has been described previously [45]. Briefly, HepG2.2.15 cells were plated in 24-well plates at a density of 10^5^ cells/well and transfected with plasmids either individually or in combination or with a nonspecific control siRNA. After being transfected for 24 h or 48 h, cell proliferation (or cellular toxicity) was analyzed by an MTT assay. For each MTT assay, the medium in each well was replaced with 400 μl of medium containing MTT at 0.5 μg/μl. After 4 h of incubation, the MTT-containing medium was removed, 400 μl of DMSO were added to each well, and the plate was agitated for 10 min in the dark to dissolve the MTT-formazan crystals. Sample absorbance was measured at 570 nm. The above experiments were performed in triplicate and the results are presented as the mean±SD.

### Assay of HEK293, T98G cells and HepG2.2.15 cells for IFN-α, IFN-β and TNF-α

To evaluate the potential effect of siRNAs on innate IFN response in human cells, HEK293, T98G cells and HepG2.2.15 cells were respectively transfected with pU6, S1, S2, S3, siHsc70, siEGFP and the positive control 0.8 μg poly (I:C) (sigama, USA), and then cell culture supernatants were harvested 48 h post-transfection to detect cytokine. The concentrations of IFN-α, IFN-β and TNF-α in cell culture supernatants from transfected cells were determined by ELISA. Meanwhile, the mRNA concentrations of IFN-α, IFN-β and TNF-α were analyzed by RT-PCR in every three cell lines. The cDNA was used as the template for quantitative RT-PCR amplification with the SYBR RT-PCR Kit. The method of extracting RNA and the reverse transcription program was the same as mentioned above. Three pairs of primers used respectively for RT-PCR were: 5’-TTTGCTTTACTGGTGGCC-3’(sense) 5’-GGGATGGTTTCAGCCTTT-3’(antisense) for IFN-α; 5’-ACAGGTAGTAGGCGACAC-3’(sense) 5’-GTTCTGGAGCATCTCATAG -3’(antisense) for IFN-β; 5’-AAGCAACAAGACCACCACTTCG-3’(sense) 5’- TCTCCAGATTCCAGATGTCAGGG-3’(antisense) for TNF-α. The thermocycling programs of quantitative RT-PCR were the same except the annealing temperature with 62°C for IFN-α, 60°C for IFN-β, and 59°C for TNF-α. GAPDH was used as an internal control, and the primers employed were the same as the primers for RT-PCR described above. Assays were performed in triplicate independent experiments.

### Statistical analysis

Statistical analysis was performed with Excel and with SPSS software (version 11.0; Chicago, IL). All comparative analyses were made using two-tailed hypothesis tests and Student’s *t*-test (*P* values of 0.05 or less were regarded as significant difference).

## Abbreviations

HBV: Hepatitis B virus; HCC: Hepatocellular carcinoma; RNAi: RNA interference; shRNA: Short hairpin RNA; siRNA: Small interfering RNA; siHsc70: Heat shock cognate 70-specific siRNA; RISC: RNA-induced silencing complex; PCR: Polymerase chain reaction; RT-PCR: Quantitative real-time reverse transcription-PCR; RT-PCR: Quantitative real-time PCR; IFN-α: Interferon alpha; IFN-β: Interferon beta; TNF-α: Tumor necrosis factor alpha; ISG: IFN-stimulated gene; TLR: Toll-like receptor; CMV: Cytomegalo virus; EGFP-siRNA: pU6-siRNA targeting EGFP; poly (I:C): polyinosinic acid:polycytidilic acid.

## Competing interests

The authors declare that they have no competing interests. Additional files are available at *Virology Journal’s* website.

## Authors’ contributions

ZQB was responsible for the experiments. ZQB, and ZXZ designed research. ZQB, AX, MMC, MQL, SL, YJ, WYY, and ZTQ performed experiments. ZQB and AX wrote the paper. All authors read and approved the final manuscript.

## Supplementary Material

Additional file 1 **Figure S1.** Schematic diagrams of shRNA-expressing cassette, EGFP reporter system, target constructs, and target viral mRNA. (A) An inverted repeat corresponding to each of the target sequences in the HBV genome was inserted under the control of pU6 and a transcriptional termination signal of five Ts. As a result, transcription of the shRNA-coding insert could be driven by pU6. The synthesized RNAs should therefore fold back to form two types of shRNAs that are finally processed into the putative siRNAs. (B) Diagram of the reporter system. To provide a reporting system for evaluating the gene-silencing efficacy of siRNAs, the DNA of HBVS was cloned into pEGFP-N1 and pcDNA3.1B (⇀) vectors as described in Materials and Methods. (C) The HBV genome contains four overlapping open reading frames. The arrows above show the sites targeted by HBVS-specific shRNAs. Click here for file

Additional file 2 **Figure S2.** Effect of siRNAs on the expression of HBV surface open reading frame in HEK293 and T98G cells. (A) Fluorescence micrographs of cells transfected with reporter plasmids and cotransfected with either the corresponding or non-corresponding siRNA with Lipofectamine ^TM^ 2000 (Invitrogen). At 24 hrs after transfection, the cells were observed with an Olympus BH-2 microscope, and representative bright-field images (left column) and relative fluorescent-field images (right column) were recorded by fourfold amplification. (B) Flow cytometry analysis of siRNA-mediated gene silencing of EGFP. EGFP expression in cells cotransfected with (a) pEGFP-N1 vector; ( b) pEGFP-N1 and siEGFP; (c) pEGFP-N1 and pU6; (d) pEGFP-N1 and S3(heterologous siRNAs) . The mean fluorescence intensity of control siRNA was taken as 100% and adopted as control. Data represent means±SD from three independent experiments carried out in triplicate. Click here for file

Additional file 3 **Figure S3.** (A) siRNA1 target sequences in various subtype sequences of HBV genome selected for homologous sequential analysis. (B) siRNA2 target sequences in various subtype sequences of HBV genome selected for homologous sequential analysis. Click here for file

Additional file 4 **Figure S4.** siRNA2 target sequences in various subtype sequences of HBV genome selected for homologous sequential analysis. Click here for file
