# Supplementary material for: Anti-HBV efficacy of combined siRNAs targeting viral gene and heat shock cognate 70
Source: Virol J. 2012 Nov 16;9:275. doi: 10.1186/1743-422X-9-275 (PMC3534549; doi:10.1186/1743-422X-9-275)
Supplement: Additional file 1 — Figure S1. Schematic diagrams of shRNA-expressing cassette, EGFP reporter system, target constructs, and target viral mRNA. (A) An inverted repeat corresponding to each of the target sequences in the HBV genome was inserted under the control of pU6 and a transcriptional termination signal of five Ts. As a result, transcription of the shRNA-coding insert could be driven by pU6. The synthesized RNAs should therefore fold back to form two types of shRNAs that are finally processed into the putative siRNAs. (B) Diagram of the reporter system. To provide a reporting system for evaluating the gene-silencing efficacy of siRNAs, the DNA of HBVS was cloned into pEGFP-N1 and pcDNA3.1B (⇀) vectors as described in Materials and Methods. (C) The HBV genome contains four overlapping open reading frames. The arrows above show the sites targeted by HBVS-specific shRNAs. [file 1743-422X-9-275-S1.doc]

**A**


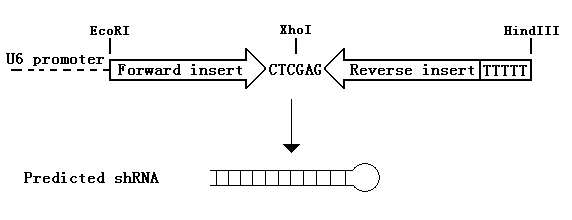


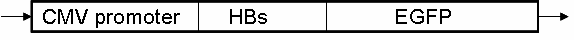
**B**


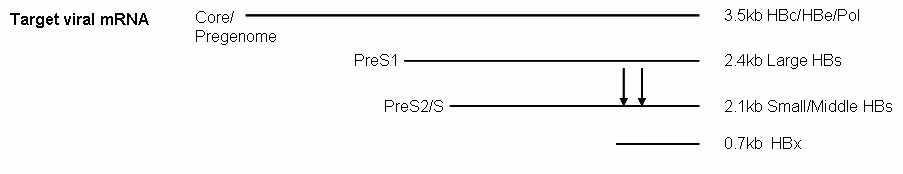
**C**

**Additional file 1** **Figure S1.**
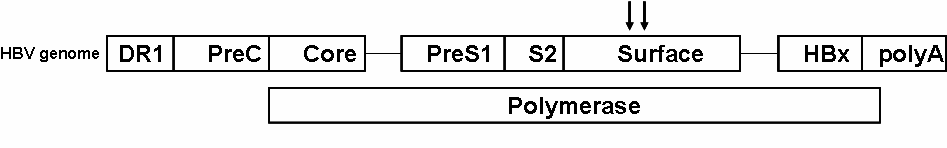
**Schematic diagrams of shRNA-expressing cassette, EGFP reporter** **system, target constructs, and target viral mRNA.** (**A**) An inverted repeat corresponding to each of the target sequences in the HBV genome was inserted under the control of pU6 and a transcriptional termination signal of five Ts. As a result, transcription of the shRNA-coding insert could be driven by pU6. The synthesized RNAs should therefore fold back to form two types of shRNAs that are finally processed into the putative siRNAs. (**B**) Diagram of the reporter system. To provide a reporting system for evaluating the gene-silencing efficacy of siRNAs, the DNA of HBV S was cloned into pEGFP-N1 and pcDNA3.1B (一) vectors as described in Materials and Methods. (**C**) The HBV genome contains four overlapping open reading frames. The arrows above show the sites targeted by HBVS-specific shRNAs.
