# Supplementary material for: Anti-HBV efficacy of combined siRNAs targeting viral gene and heat shock cognate 70
Source: Virol J. 2012 Nov 16;9:275. doi: 10.1186/1743-422X-9-275 (PMC3534549; doi:10.1186/1743-422X-9-275)
Supplement: Additional file 2 — Figure S2. Effect of siRNAs on the expression of HBV surface open reading frame in HEK293 and T98G cells. (A) Fluorescence micrographs of cells transfected with reporter plasmids and cotransfected with either the corresponding or non-corresponding siRNA with Lipofectamine TM 2000 (Invitrogen). At 24 hrs after transfection, the cells were observed with an Olympus BH-2 microscope, and representative bright-field images (left column) and relative fluorescent-field images (right column) were recorded by fourfold amplification. (B) Flow cytometry analysis of siRNA-mediated gene silencing of EGFP. EGFP expression in cells cotransfected with (a) pEGFP-N1 vector; ( b) pEGFP-N1 and siEGFP; (c) pEGFP-N1 and pU6; (d) pEGFP-N1 and S3(heterologous siRNAs) . The mean fluorescence intensity of control siRNA was taken as 100% and adopted as control. Data represent means±SD from three independent experiments carried out in triplicate. [file 1743-422X-9-275-S2.doc]

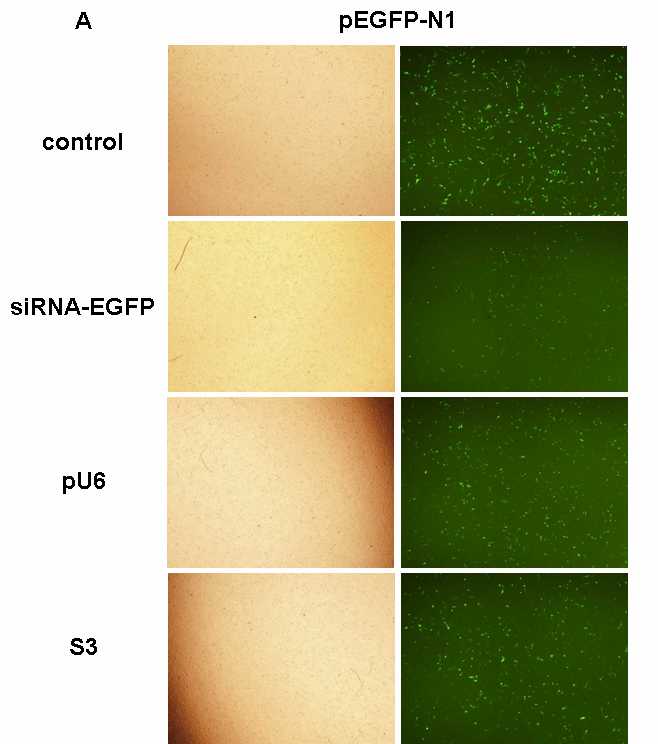


**B**


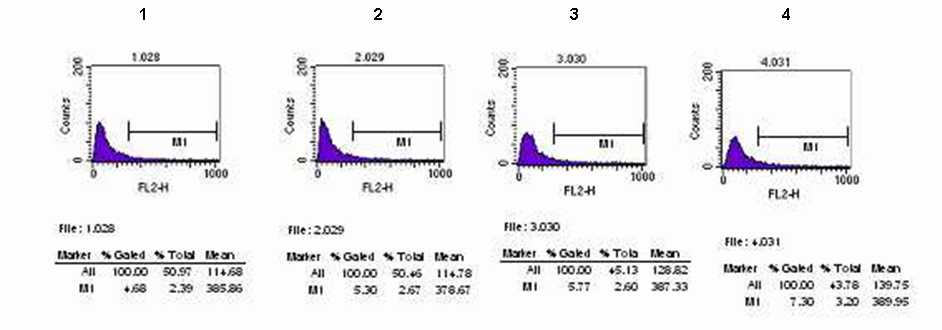


**(a)**

**(b)**

**(c)**

**(d)**

**Additional file 2 Figure S2. Effect of siRNAs on the expression of HBV surface open reading frame in HEK293 and T98G cells. (A)** Fluorescence micrographs of cells transfected with reporter plasmids and cotransfected with either the corresponding or non-corresponding siRNA with Lipofectamine ^TM^ 2000 (Invitrogen). At 24 hrs after transfection, the cells were observed with an Olympus BH-2 microscope, and representative bright-field images (left column) and relative fluorescent-field images (right column) were recorded by fourfold amplification. **(B)** Flow cytometry analysis of siRNA-mediated gene silencing of EGFP. EGFP expression in cells cotransfected with (a) pEGFP-N1 vector; ( b) pEGFP-N1 and siEGFP; (c) pEGFP-N1 and pU6; (d) pEGFP-N1 and S3(heterologous siRNAs) . The mean fluorescence intensity of control siRNA was taken as 100% and adopted as control. Data represent means±SD from three independent experiments carried out in triplicate.
