# Supplementary material for: Anti-HBV efficacy of combined siRNAs targeting viral gene and heat shock cognate 70
Source: Virol J. 2012 Nov 16;9:275. doi: 10.1186/1743-422X-9-275 (PMC3534549; doi:10.1186/1743-422X-9-275)
Supplement: Additional file 3 — Figure S3. (A) siRNA1 target sequences in various subtype sequences of HBV genome selected for homologous sequential analysis. (B) siRNA2 target sequences in various subtype sequences of HBV genome selected for homologous sequential analysis. [file 1743-422X-9-275-S3.doc]

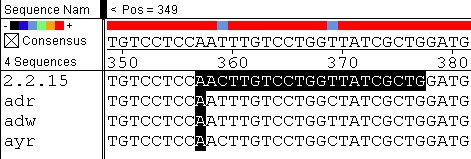


**Additional file 3** Figure S3 siRNA1 (S1) target sequences in various subtype sequences of HBV genome selected for homologous sequential analysis.


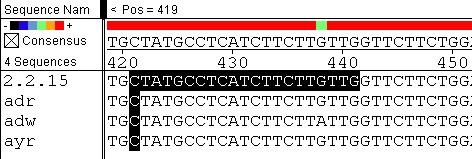


**Additional file 4** Figure S4 siRNA2 (S2) target sequences in various subtype sequences of HBV genome selected for homologous sequential analysis.
