# Supplementary material for: Anti-HBV efficacy of combined siRNAs targeting viral gene and heat shock cognate 70
Source: Virol J. 2012 Nov 16;9:275. doi: 10.1186/1743-422X-9-275 (PMC3534549; doi:10.1186/1743-422X-9-275)
Supplement: Additional file 4 — Figure S4. siRNA2 target sequences in various subtype sequences of HBV genome selected for homologous sequential analysis. [file 1743-422X-9-275-S4.doc]

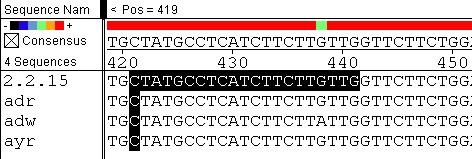


**Additional file 4** Figure S4 siRNA2 (S2) target sequences in various subtype sequences of HBV genome selected for homologous sequential analysis.
